# 3-Iodothyronamine Activates a Set of Membrane Proteins in Murine Hypothalamic Cell Lines

**DOI:** 10.3389/fendo.2018.00523

**Published:** 2018-09-11

**Authors:** Julia Bräunig, Stefan Mergler, Sabine Jyrch, Carolin S. Hoefig, Mark Rosowski, Jens Mittag, Heike Biebermann, Noushafarin Khajavi

**Affiliations:** ^1^Charité – Universitätsmedizin Berlin, Corporate Member of Freie Universität Berlin, Humboldt-Universität zu Berlin, and Berlin Institute of Health, Berlin, Germany; ^2^Institute of Experimental Pediatric Endocrinology, Berlin, Germany; ^3^Klinik für Augenheilkunde, Charité – Universitätsmedizin Berlin, Corporate Member of Freie Universität Berlin, Humboldt-Universität zu Berlin, and Berlin Institute of Health, Berlin, Germany; ^4^Institute of Experimental Endocrinology, Charité – Universitätsmedizin Berlin, Corporate Member of Freie Universität Berlin, Humboldt-Universität zu Berlin, and Berlin Institute of Health, Berlin, Germany; ^5^Department of Cell & Molecular Biology, Karolinska Instituet, Stockholm, Sweden; ^6^Department Medical Biotechnology, Institute of Biotechnology, Technical University of Berlin, Berlin, Germany; ^7^University of Lübeck – Center of Brain Behavior and Metabolism, Lübeck, Germany

**Keywords:** 3-T_1_AM, signaling, hypothalamus, GPCR, TRP Channel

## Abstract

3-Iodothyronamine (3-T_1_AM) is an endogenous thyroid hormone metabolite. The profound pharmacological effects of 3-T_1_AM on energy metabolism and thermal homeostasis have raised interest to elucidate its signaling properties in tissues that pertain to metabolic regulation and thermogenesis. Previous studies identified G protein-coupled receptors (GPCRs) and transient receptor potential channels (TRPs) as targets of 3-T_1_AM in different cell types. These two superfamilies of membrane proteins are largely expressed in tissue which influences energy balance and metabolism. As the first indication that 3-T_1_AM virtually modulates the function of the neurons in hypothalamus, we observed that intraperitoneal administration of 50 mg/kg bodyweight of 3-T_1_AM significantly increased the c-FOS activation in the paraventricular nucleus (PVN) of C57BL/6 mice. To elucidate the underlying mechanism behind this 3-T_1_AM-induced signalosome, we used three different murine hypothalamic cell lines, which are all known to express PVN markers, GT1-7, mHypoE-N39 (N39) and mHypoE-N41 (N41). Various aminergic GPCRs, which are the known targets of 3-T_1_AM, as well as numerous members of TRP channel superfamily, are expressed in these cell lines. Effects of 3-T_1_AM on activation of GPCRs were tested for the two major signaling pathways, the action of Gα_s_/adenylyl cyclase and G_i/o_. Here, we demonstrated that this thyroid hormone metabolite has no significant effect on G_i/o_ signaling and only a minor effect on the Gα_s_/adenylyl cyclase pathway, despite the expression of known GPCR targets of 3-T_1_AM. Next, to test for other potential mechanisms involved in 3-T_1_AM-induced c-FOS activation in PVN, we evaluated the effect of 3-T_1_AM on the intracellular Ca^2+^ concentration and whole-cell currents. The fluorescence-optic measurements showed a significant increase of intracellular Ca^2+^ concentration in the three cell lines in the presence of 10 μM 3-T_1_AM. Furthermore, this thyroid hormone metabolite led to an increase of whole-cell currents in N41 cells. Interestingly, the TRPM8 selective inhibitor (10 μM AMTB) reduced the 3-T_1_AM stimulatory effects on cytosolic Ca^2+^ and whole-cell currents. Our results suggest that the profound pharmacological effects of 3-T_1_AM on selected brain nuclei of murine hypothalamus, which are known to be involved in energy metabolism and thermoregulation, might be partially attributable to TRP channel activation in hypothalamic cells.

## Introduction

3-iodothyronamine (3-T_1_AM) is a decarboxylated and deiodinated derivative of thyroid hormones (1, 2). Although several studies detected 3-T_1_AM in human blood (3, 4), the mechanism of physiological action of this compound in the human body remains undefined. Administration of 3-T_1_AM in rodents blocks the hypothalamic—pituitary—thyroid axis and results in concentration-dependent reversible effects on body temperature, energy metabolism, cardiac and neurological functions (1, 5). Previous observations in rodents demonstrated the accumulation of 3-T_1_AM in different tissues such as kidney, liver, muscles, and brain (6). In mice, after administration of a radioisotope labeled [^125^I]-3-T_1_AM, ^125^I was detected in the brain (6). In rats, site-directed injections of 3-T_1_AM into the locus coeruleus elicits significant neuronal firing rate changes in the selected brain nuclei such as the paraventricular nucleus (PVN) of the hypothalamus (7). Interestingly, the target areas of 3-T_1_AM in the brain nuclei are mostly involved in energy homeostasis, arousal, and memory retrieval (8–11). Presumably, different effects of 3-T_1_AM, such as anapyrexia and food consumption might be centrally mediated via the hypothalamus (12–14). Due to the profound pharmacological effects of 3-T_1_AM and its accumulation in the selected tissue, numerous studies over the last years have been devoted to investigating the signaling property of this thyroid hormone metabolite.

The first target of 3-T_1_AM is the trace amine associated receptor 1 (TAAR1), a trace amine-activated G protein-coupled receptor (GPCR) (1). 3-T_1_AM induces Gα_s_/adenylyl cyclase signaling in rodent TAAR1 and human *TAAR1*-transfected cells (1). Additionally, different studies described several other GPCRs as 3-T_1_AM targets, mainly *in vitro* and in overexpressing systems. These GPCRs belong to the group of aminergic GPCRs (15) such as the α-2A-adrenergic receptor (ADRA2A (16), the β2-adrenergic receptor (ADRB2) (17), the muscarinergic receptor 3 (CHRM3) (18), or the serotonin receptor 1b (5-HT1b) (19). Moreover, 3-T_1_AM modulates calcium and potassium homeostasis through an intracellular calcium channel, known as “ryanodine receptor” in adult rat cardiomyocytes (20).

Recent studies identified non-selective cation channels such as the transient receptor potential channel melastatin 8 (TRPM8) and the transient receptor potential vanilloid 1 (TRPV1) as novel targets of 3-T_1_AM (21–23). Classically, TRPM8 is known as a cold and menthol receptor and is a temperature-sensitive receptor in excitable cells (24). Its activation induces a depolarization of the cell membrane leading to action potentials. The same function principle applies to TRPV1, which is known as a heat- and capsacin receptor (25). Together, these properties implicate these TRPs as possible transducers of cold or warm stimuli not only within the hypothalamus (26), but also in keratintocytes of human skin (27) and neurons on human corneal nerve fibers (28, 29).

Different studies demonstrated that TRPs are the major downstream effectors of GPCRs and the signaling cascades that emanate from the activation of GPCR evoke TRP channel activity (30, 31). There is a wide distribution of TRPs in tissues that influence energy homeostasis and thermoregulation. Expression of TRPs in various tissues such as hypothalamus, peripheral sensory neurons, gastrointestinal tract, liver, adipocytes, and ocular tissues strongly suggest the possible role these ion channels play in energy balance and metabolism as well as thermoregulation (32–37). Modulation of TRPs via 3-T_1_AM raises the question of what could be the 3-T_1_AM-induced signalosome and whether there is a link between stimulatory effects of 3-T_1_AM in tissues that pertain to metabolic- and/or thermo-regulation and TRPs.

Here, we identified the stimulatory effect of 3-T_1_AM in murine hypothalamic nuclei and explored the underlying mechanism behind this effect in murine hypothalamic cell lines. The results of this study show a stimulatory effect of 3-T_1_AM on Ca^2+^ mobilization and whole-cell currents in murine hypothalamic cells and that this effect is associated with TRPM8 activation.

## Methods

### Mice experiments

#### Immunohistochemistry

In collaboration with the Karolinska Institute, Sweden, C57BL/6J mice (4 in each group) were i.p. injected with 50 mg/kg body weight 3-T_1_AM solved in 60% DMSO and 40% PBS, control mice with 60% DMSO and 40% PBS (volume of injection was 5 μl/g body weight). After 60 min, animals were transcardially perfused with PBS and 10% formalin (European Community Council Directives (86/609/EEC) and approved by Stockholm's Norra Djurförsöksetiska Nämnd). Fixated murine brains were successively incubated in 10, 20, and 30% sucrose solution over several days. Brains were cut at a cryotom into 30 μm slices and placed in a 48 well plate filled with PBS. Slices were blocked with a blocking buffer (TBS, 0.25% gelatin from porcine skin and 0.5% triton X100) for 2 h, subsequently incubated with a c-FOS antibody, rabbit anti mouse (1:200; Santa Cruz Biotechnology, Santa Cruz, CA, USA) over night at 4°C and finally with an Alexa Fluor 549 antibody, goat anti rabbit (1:200; Jackson ImmunoResearch) for 2 h at room temperature. Between each step, the slides were washed 3 × 1 min with TBS (tris-buffered saline). The brain slices were placed on glass slides and mounted with VectaShield containing DAPI (Vector Laboratories, Burlingame, CA, USA). Pictures were taken with a Keyence BZ-9000 microscope (20 × magnification) and optimized with the ImageJ software. C-FOS positive cells were counted with the ImageJ software using two brain slides of each animal for each brain loci.

### Cell culture and mRNA isolation

GT1-7 (mouse hypothalamic gonadotropin-releasing-hormone neuronal cell line) were purchased from MERK, (38). mHypoE-N39 (N39) and mHypoE-N41 (N41), both embryonic mouse hypothalamic cell lines, were acquired from Cedarlane, established by Belsham et al. (39). A screening profile of neuronal markers of all three cell lines can be viewed here (https://www.cedarlanelabs.com/Products/Listing/Hypothalamic). The cells were cultured in Dulbecco's modified Eagle's medium (DMEM, Biochrom GmbH, Berlin, Germany), supplemented with 10% fetal calf serum (FCS) and 1% penicillium/streptavidin at 37°C in humidified air containing 5% CO_2_.

Cells were seeded in T75 flask and grown to 80% confluence. Cells were harvested in three different passages, spanning from passage three to passage six. Total mRNA was isolated by chloroform/phenol extraction, a DNase digestion was performed and samples were stored at −80°C.

### Quantitative PCR to determine GPCR and TRP channel expression profiles

A quantitative PCR (qPCR) was performed to determine the expression level of several GPCRs and TRPs. QPCR primers including their efficiency are listed in Supplemental Table 1. *Pgk1* was chosen as the reference gene, as previously recommended (40). First, mRNA was transcribed into cDNA by the Ominscript RT kit (Qiagen) using random hexameres (Applied Biosystems) and Oligo dTs (Promega, Madison, USA). Absolute QPCR Mix, SYBR Green, no Rox (Thermo Scientific, Germany) was used for qPCR on a Stratagene Mx3000P System using 100 nM per primer. PCR reaction underwent an initial cycle at 95°C for 15 min followed by 42 cylces at 95°C for 15s, primer specific annealing step 60°C for 30 s and a elongation step 72°C for 45 s, and elongation at 72°C for 7 min and finally temperature holding at 4°C. Melting curve analysis was performed to confirm the specificity of the PCR reaction. Data was processed using the Δct method. We used the slope of a standard curve to determine the amplification efficiency for each primer pair (efficiency = 10^−1/slope^). Each of the three passages of every cell line was measured in duplicates together with a sample without reverse transcription to exclude genomic DNA contamination.

### Gα_s_ and G_i/o_ signaling of endogenous expressed GPCRs in hypothalamic cell lines

Gα_s_ and G_i/o_ signaling were determined by measuring cAMP accumulation using the AlphaScreen technology (PerkinElmer Life Science, Boston, MA, USA) as previously described (15). Cells were cultured in poly-L-lysine (Biochrom GmbH, Berlin, Germany) coated 96-well plates (1 × 10^−4^ cells / well). Seventy-two hours after seeding, stimulation was performed by means of using a stimulation buffer (138 mM NaCl, 6 mM KCl, 1 mM [MgCl${}_{2}^{*}$6H_2_O], 5.5 mM glucose, 20 mM HEPES, 1 mM [CaCl${}_{2}^{*}$2H_2_O], 1 mM IBMX). For Gα_s_ signaling, cells were incubated for 45 min with either 3-T_1_AM (Santa Cruz Biotechnology, Dallas, TX, USA), serotonin (5-HT, Sigma-Alderich, St. Louis, MO, USA), norepinephrine (NorEpi, Sigma-Alderich, St. Louis, MO, USA), isoproterenol (ISOP, Sigma-Alderich, St. Louis, MO, USA), or phenethylamine (PEA, Sigma-Alderich, St. Louis, MO, USA) in a concentration of 10 μM or only stimulation buffer to monitor the basal cAMP content. 3-T_1_AM was diluted from a 10 mM stock solution using DMSO as solvent. H_2_O was used as solvent for serotonin and norepinephrin and PBS with 0.1% BSA was used as the solvent for isoproterenol and phenethylamine. For G_i/o_ pathway examination, cells were additionally stimulated with 50 μM forskolin (FSK, AppliChem GmbH, Darmstadt, Germany) to activate the adenylyl cyclase for a total of 45 min. Afterwards, cells were lysed at 4°C on a shaking platform. Intracellular cAMP accumulation was determined by a competitive immunoassay based on the AlphaScreen assay kit according to the manufacturer's instructions and measured using a Berthold Microplate Reader (Berthold Technologies GmbH & Co. KG, Bad Wildbad, Germany). Cyclic AMP concentrations were normalized to protein contents, which was measured with the Pierce BCA Protein Assay Kit (Thermo Scientific, Germany).

### Determination of intracellular Ca^2+^ concentration

To monitor time-dependent changes in intracellular free Ca^2+^ levels ([Ca^2+^]_i_) in single-cells, cells were pre-incubated with culture medium containing fura-2/AM (2 μM) for ~30 min at 37°C. Loading was stopped with a Ringer-like (control) solution containing: 150 mM NaCl, 6 mM CsCl, 1 mM MgCl_2_, 10 mM glucose, 10 mM HEPES, and 1.5 mM CaCl_2_ at pH 7.4. Where a blocker was used, pre-incubation was performed 30 min before the measurement. Fluorescence measurements were performed on the stage of an invert microscope (Olympus BW50WI) and a camera (Olympus XM-10) in connection with a LED-Hub (Omikron, Rodgau-Dudenhoven, Germany). Fura-2/AM fluorescence was excited at 340 and 380 nm alternatingly and emission was detected from small cell clusters every 4 s at 510 nm. Results are shown as mean traces of f_340nm_/f_380nm_ ± SEM. Drugs were dissolved in dimethyl sulfoxide (DMSO) to obtain a stock solution and diluted in Ringer-like solution to obtain a working concentration which did not exceed 0.1%. For image acquisition and data evaluation, the life science imaging software cellSens was used (Olympus, Hamburg, Germany).

### Planar patch-clamp recordings

For electrophysiological recordings, the semi-automated planar patch-clamp technique was used as previously described (41). Whole-cell currents were evaluated in conjunction with an EPC10 amplifier and PatchMaster acquisition software (HEKA, Lambrecht, Germany) as well as PatchControl software (Nanion, Munich, Germany). For recording, 5 μl of an internal-like solution was applied to the internal side of the microchip. The internal solution contained: 50 mM CsCl, 10 mM NaCl, 2 mM MgCl_2_, 60 mM CsF, 20 mM EGTA, and 10 mM HEPES, pH 7.2 and osmolarity 288 mOsM. Cs in the internal solution blocks potassium channel activity. A single cell suspension was added to an external solution of the following composition: 140 mM NaCl, 4 mM KCl, 1 mM MgCl_2_, 2 mM CaCl_2_F, 5 mM D-glucose monohydrate, and 10 mM HEPES, pH 7.4 and osmolarity 298 mOsM. Whole-cell currents were recorded using a ramp protocol ranging between −60 to +130 mV for 500 milliseconds. The mean membrane capacitance of N41 cells was 9 pF ± 1 pF (*n* = 10). Mean access resistance was 15 ± 1 MΩ (*n* = 10). The holding potential (HP) was set to 0 mV in order to eliminate any possible contribution of VDCCs or sodium channels. All plots were generated with SigmaPlot software version 12.5 (Systat, San Jose, California, USA).

### Data evaluation and statistics

GraphPad Prism 6.0 (GraphPad software, San Diego, Calif., USA) was chosen for visualization and data analysis. Data are shown as means ± SEM of independent experiments. Statistical analysis was carried out using one-way and two-way ANOVA, followed by Sidak correction. For Ca^2+^ imaging, statistical significance was determined by an unpaired *t*-test with Welch's correction. In figure legends, the number of experiments and the type of comparison are given. Statistical significance was defined as ^*^p ≤ 0.05, ^**^p ≤ 0.01, ^***^p ≤ 0.001, and ^****^p ≤ 0.0001.

## Results

### Intraperitoneal injection of 3-T_1_AM results in the activation of PVN neurons in C57BL/6 mice

**Figure 1:**
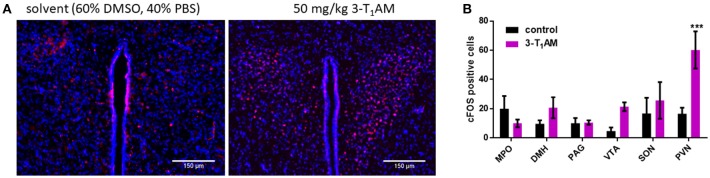
Staining of c-FOS activated neurons after 1 h of i.p. injection of 50 mg/kg 3-T_1_AM. After intraperitoneal injection of the C57BL/6J mice with either 3-T_1_AM or solvent (60% DMSO/40% PBS), brains were frozen and cryosectioned and stained against c-FOS and DAPI. (**A**) In comparison to the control mice, 3-T_1_AM-treated mice showed a strong c-FOS staining in the PVN. All pictures were taken with a 20 × objective. (**B**) c-FOS positives cells were counted in the respective nuclei. Only 3-T_1_AM treated animals (*n* = 4) showed an increase in c-FOS activity in the PVN. For statistics a two-way ANOVA was performed, followed by a Sidak correction; ***p ≤ 0.001.

Previous studies demonstrated the accumulation and stimulatory effects of 3-T_1_AM in selected brain nuclei such as locus coeruleus and PVN of the hypothalamus (6, 7). However, the exact role of this thyroid hormone metabolite in the hypothalamus remains unclear. To investigate whether 3-T_1_AM is capable of activating hypothalamic neurons in these nuclei *in vivo*, we performed intraperitoneal injections of 50 mg/kg bodyweight of 3-T_1_AM or DMSO/PBS as control, six mice per group, and monitored 3-T_1_AM-induced neuron activation relative to DMSO/PBS. We used c-FOS as the marker for neuronal activity. One hour after 3-T_1_AM injection, increased c-FOS staining of distinct neurons was clearly visible in the PVN (60 ± 13 c-FOS positive cells per brain slide), while DMSO/PBS treated mice showed only few c-FOS positive neurons (16 ± 4 c-FOS positive cells per brain slide) (Figures 1A,B). 3-T_1_AM had no stimulatory effect on the medial preoptic area (MPO), the supraoptic nucleus (SON), the dorsolmedial nucleus of the hypothalamus, the periaqueductal gray (PAG) and the ventral tegmental segment (VTA) (Figure 1B and Supplemental Figure 1). The c-FOS positive cells ranged from 4 to 25 per brain slide in these brain loci.

### Expression profile of GPCRs, the main 3-T_1_AM target, in the murine hypothalamic cell lines

**Figure 2:**
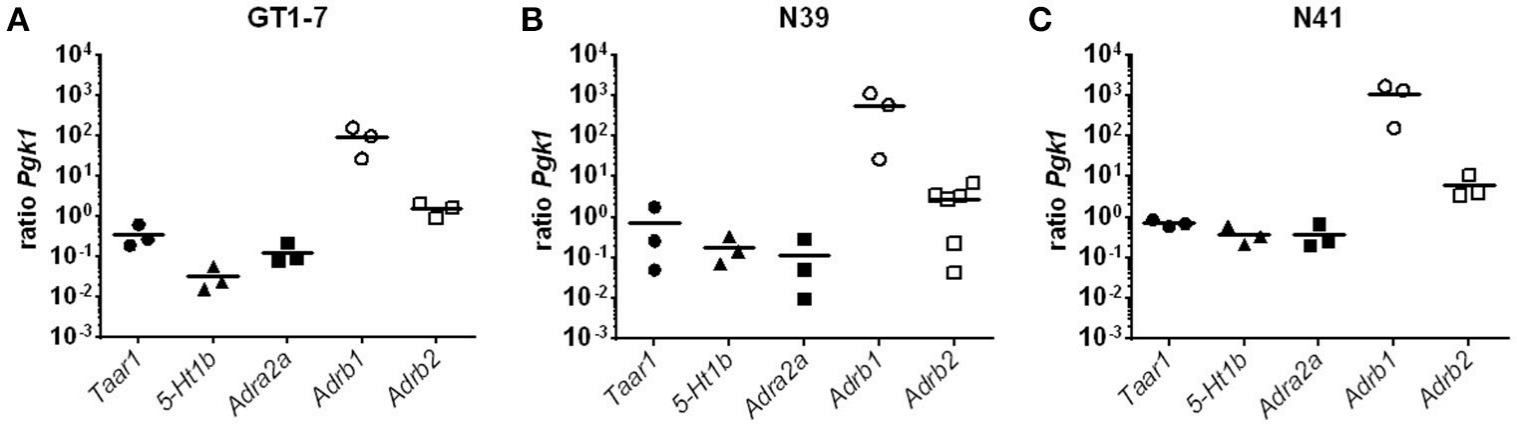
Expression pattern of GPCRs in N39, N41 and GT1-7. Results of a SYBR Green based qPCR. Graphs show the ratios between the reference gene *Pgk1* and the GPCRs *Taar1, 5-Ht1b, Adra2a, Adrb1*, and *Adrb2*. Data was pooled from (*n* = 3) measured in duplicates. (**A**) *Adrb1* and *Adrb2* have the highest expression, while *5-Ht1b* has the lowest in GT1-7 cells. (**B**) In N39 cells, *Adrb1* has the expression ratio compared to *Pgk1*, followed by *Adrb2*. *5-Ht1b* and *Adra2a* are at least abundant in N39 cells. (**C**) Among the measured GPCRs *Adrb1* has the most copies of mRNA in the transcriptome of N41 cells, once again followed by *Adrb2*. However, *Taar1, 5-Ht1b*, and *Adra2a* are rather equimolar to *Pgk1*.

To elucidate the underlying mechanism behind the stimulatory effect of 3-T_1_AM in the hypothalamus, we used three murine hypothalamic cell lines, GT1-7, mHypoE-N39 (N39), and mHypoE-N41 (N41). These cell lines are established models to study neuroendocrine mechanisms and known to express PVN-like markers (38, 42, 43). Here we demonstrated that in N41 cells, 3-T_1_AM significantly increased the c-FOS activation (Supplemental Figure 2). As GPCRs are the primary targets of 3-T_1_AM, we investigated the GPCR expression profile of the three cell lines. Here, we showed the expression of the aminergic receptors *Taar1, 5-Ht1b, Adra2a, Adrb1*, and *Adrb2* (Figure 2). Furthermore, when compared against of *Pgk1, Adrb1* noticeably had the highest expression rate among these receptors in all three cell lines with a ratio of 94 ± 38 for GT1-7, 565.6 ± 312 for N39, and 1046 ± 456 for N41. The second highest expression was detected for *Adrb2* with a ratio of 1.54 ± 0.34 for GT1-7, 2.77 ± 1.04 for N39, and 6.06 ± 2.34 for N41. The other three receptors *Taar1, 5-Ht1b*, and *Adra2a*, displayed similar expression profiles. The mRNA content in all three cell lines was lower than the reference gene *Pgk1* (Figure 2), with ratios between 0.37 ± 0.14 for *5-Ht1b* in GT1-7 and up to 0.72 ± 0.08 for *Taar1* in N41.

### 3-T_1_AM induces FSK-amplified Gα_s_ signaling in the murine hypothalamic cell lines

**Figure 3:**
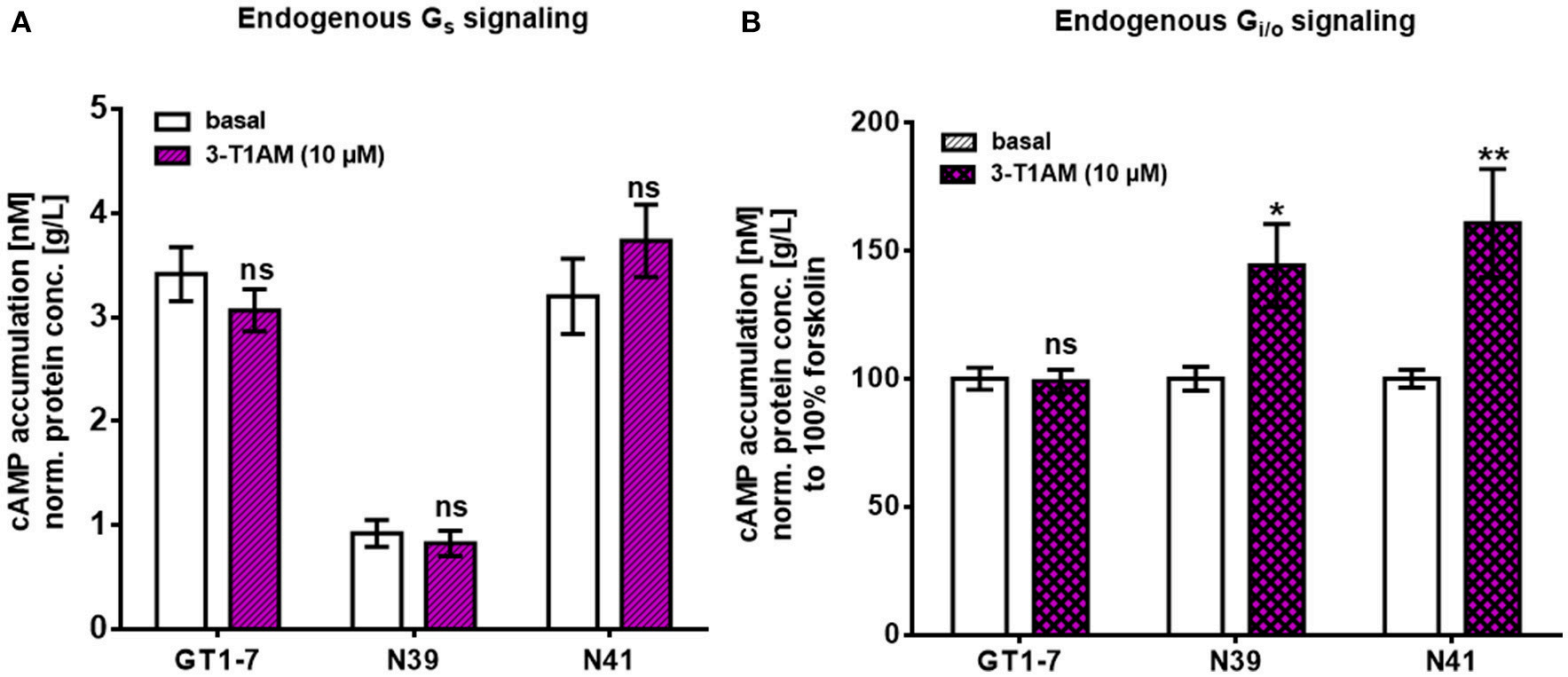
3-T1AM induces FSK-stimulated Gα_s_ signaling in murine hypothalamic cell lines. For Gα_s_ and G_i/o_, the cAMP content was measured via an AlphaScreen technology. Data are pooled from four independent assays measured in triplicates (*n* = 4). For statistics a two-way ANOVA was performed, followed by a Sidak correction. Statistics were set to *p ≤ 0.05, **p < 0.01. **(A)** Cells were stimulated with stimulation buffer or 3-T1AM in a concentration of 10^−5^ M for 45 min (*n* = 4*)*. **(B)** Cells were co-stimulated with 50 μM FSK and either stimulation buffer or 3-T1AM (10 μM) for 45 min (*n* = 4).

To investigate which pathway could contribute to 3-T_1_AM actions in the hypothalamus, we tested two major signaling cascades downstream of 3-T_1_AM GPCR targets, Gα_s_ and G_i/o_. To measure endogenous Gα_s_ signaling, we determined the cAMP enhancement. N41 and GT1-7 cells had a higher basal cAMP content with 3.2 ± 0.36 nM cAMP/g/L protein for N41 and 3.41 ± 0.26 nM cAMP/g/L protein for GT1-7, compared to N39 with 0.92 ± 0.13 nM cAMP/g/L protein (Figure 3A, *n* = 4 in triplicates, *p* < 0.001). In all cell lines, 3-T_1_AM stimulation (10 μM) did not increase cAMP concentration compared to the basal cAMP content (Figure 3A). Only NorEpi and ISOP activated an endogenous Gα_s_ signal in GT1-7 (~1.6 fold for NorEpi and ~1.7 fold for ISOP), N41 (~1.8 fold for NorEpi and ~2.1 fold for ISOP), and N39 (~3.5 fold for NorEpi and ~5.9 fold for ISOP) cells (Supplemental Figure 3). 5-HT and PEA, endogenous ligands for 5-HT1b and TAAR1, did not increase cAMP content (Supplemental Figure 3).

To determine G_i/o_ signaling, cells were incubated with FSK, an unspecific activator of the adenylyl cyclase, which increases the cellular cAMP content. It is known that activation of G_i/o_ leads to inhibition of the adenylyl cyclase and a decrease in FSK-induced cAMP content. For all three cell lines, G_i/o_ activation was not detected after stimulation with 10 μM 3-T_1_AM (Figure 3B). In addition, FSK can potentiate weak Gα_s_ signaling. Dessauer et al. showed that FSK in the presence of Gα_s_ has a higher affinity to the adenylyl cyclase, yielding in a higher cAMP accumulation (44). Here, this phenomenon emerges for N39 and N41 and 3-T_1_AM stimulation significantly increases cAMP content in FSK-treated cells (144.2 ± 16.15% for N39 and 160.77 ± 21.17% for N41, *n* = 4 in triplicates, p_N39_ = 0.0128, p_N41_ = 0.0019). The specific ligands for TAAR1, 5-HT1b, ADRA2A, ADRB1, and ADRB2 did not activate G_i/o_ signaling (Supplemental Figure 4). Collectively, in addition to an FSK-potentiated Gα_s_ activation, 3-T_1_AM had no detectable influence on cAMP content in the hypothalamic cell lines.

### Gene expression profile of TRPS in murine hypothalamic cell lines

**Figure 4:**
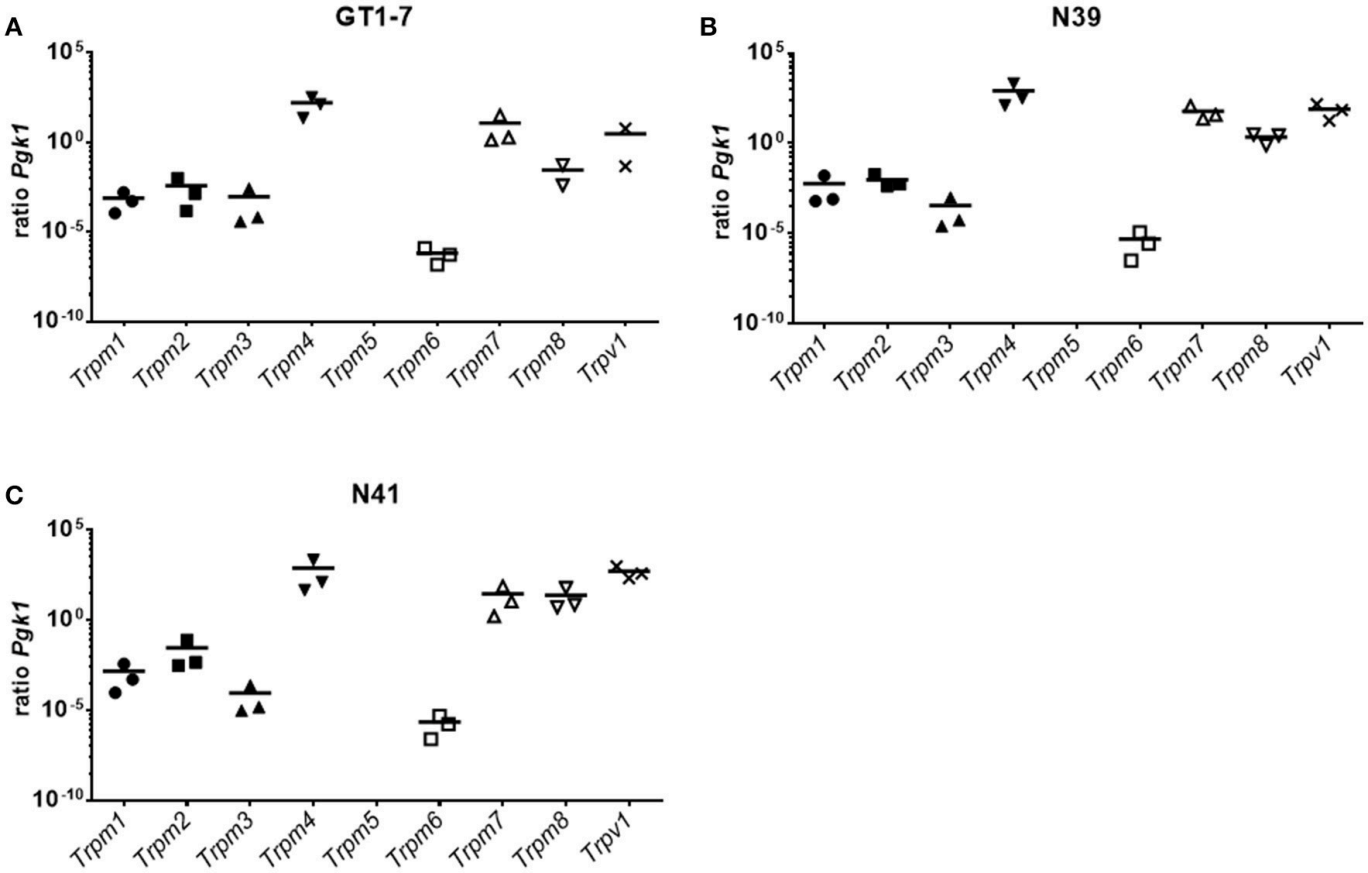
Expression pattern of TRPM channels in **(A)** GT1-7, **(B)** N39 and **(C)** N41. Results of a SYBR Green based qPCR. Graphs show the ratio between the reference gene *Pgk1* and the TRPM channels and the *Trpv1*. The three hypothalamic cell lines show a resemblance of expression pattern. *Trpm4* has always the highest expression. A cluster of *Trpm7, Trpm8*, and *Trpv1* display the second highest ratios compared to *Pgk1*. *Trpm1, Trpm2*, and *Trpm3* also form a cluster with similar expressions, however clearly lower expressed than *Trpm7, Trpm8*, and *Trpv1*. *Trpm6* is detected at with the least mRNA content of all TRPMs. *Trpm5* is not expressed in these cell lines. Data was pooled from three independent experiments measured in duplicates.

The aforementioned results demonstrated that GPCR-dependent signaling is not the sole regulator of 3-T_1_AM-induced effects on hypothalamic cells. Previous studies identified several members of TRPs as new targets for 3-T_1_AM (21–23). Here, we measured the gene expression levels of the TRPM subfamily and TRPV1 in three hypothalamic cell lines. The qPCR data show that none of the hypothalamic cell lines expressed *Trpm5* (Figure 4). Contrary to this, in GT1-7 cells, *Trpm4* was the highest expressed TRP channel with a ratio of 159 ± 89 compared to *Pgk1*, followed by *Trpm7* with a ratio of 11.7 ± 10.1 to *Pgk1*. *Trpv1* had a ratio of 2.96 ± 2.91 and *Trpm8* of 0.03 ± 0.02 to *Pgk1*. *Trpm1* was expressed with a ratio of 0.00075 ± 0.00045 to *Pgk1*. *Trpm6* had the lowest expression with a ratio of 0.00000064 ± 0.00000031 compared to *Pgk1* (Figure 4A). In N39 cells, *Trpm4* was also the highest expressed TRP channel with a ratio of 831 ± 617 to *Pgk1*, followed by *Trpv1* with a ratio of 79 ± 38 to *Pgk1*. *Trpm7* had a ratio of 59 ± 29 and *Trpm8* a ratio of 2.19 ± 0.72. *Trpm1, Trpm2*, and *Trpm3* expression ratios laid between a ratio of 0.029 ± 0.025 to *Pgk1* for *Trpm2* and *Trpm3* with a ratio of 0.000089 ± 0.000076 to *Pgk1*. The mRNA content was comparable to GT1-7 cells, with *Trpm6* having the lowest expression with a ratio of 4.95 ± 3.48 compared to the reference gene (Figure 4B). N41 cells exhibited a similar expression pattern of TRP channels as GT1-7 and N39 cell lines. *Trpm4* (ratio to *Pgk1* 759 ± 674) and *Trpv1* (ratio to *Pgk1* 514 ± 226) were the highest expressed genes, followed by *Trpm7* (ratio to *Pgk1* 29 ± 23) and *Trpm8* (24 ±18). *Trpm2* and *Trpm1* were lower expressed with ratios of 0.029 ± 0.025 and 0.0014 ± 0.0011 compared to the reference gene. *Trpm3* and *Trpm6* were least expressed in the transcriptome of N41 cells with ratios to *Pgk1* of 0.000089 ± 0.000076 and 0.0000022 ± 0.0000013 (Figure 4C).

### 3-T_1_AM increases intracellular Ca^2+^ concentration and whole-cell currents in murine hypothalamic cell lines

**Figure 5:**
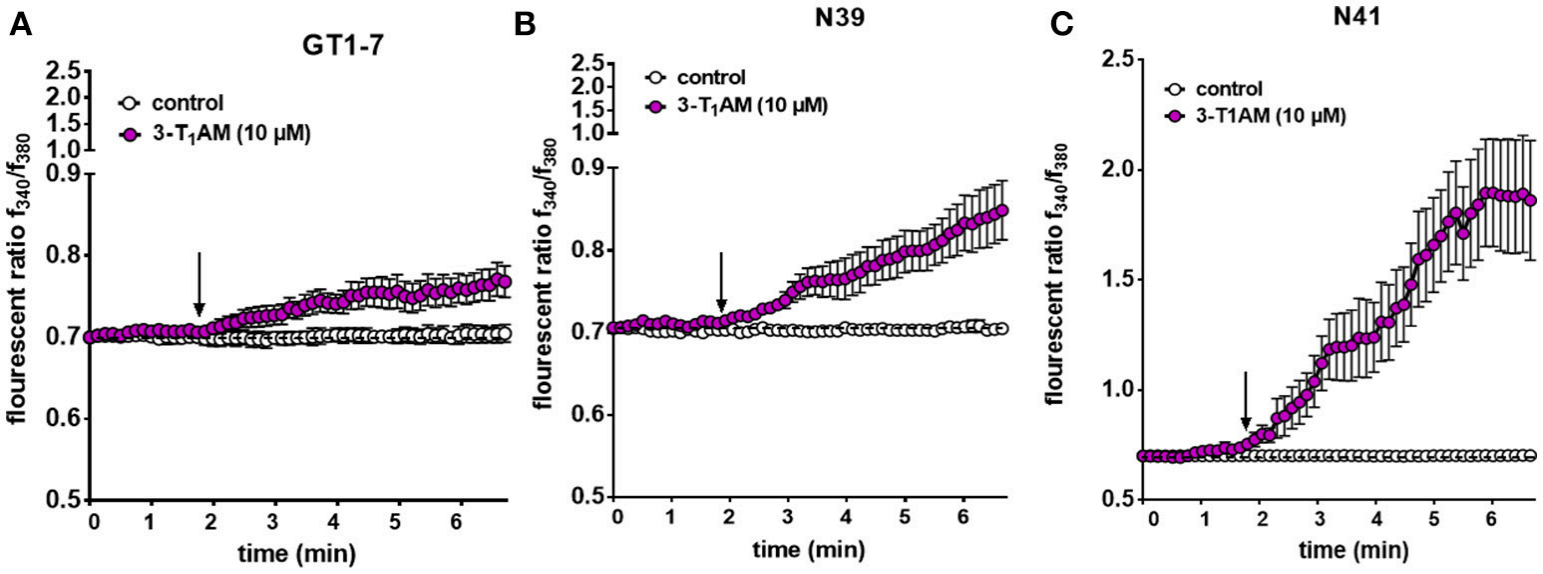
3-T_1_AM induces Ca^2+^ influx in GT1-7, N39 and N41 cell lines. Changes in cytosolic free Ca^2+^ are depicted as the ratio of the fluorescence induced by the excitation wavelength at 340 and 380 nm. 10 μM 3-T_1_AM induces an increase on intracellular Ca^2+^ concentration in **(A)** GT1-7 (*n* = 10), **(B)** N39 (*n* = 15) and **(C)** N41 (*n* = 15) cells. Notably, 10 μM 3-T_1_AM induces a significantly larger increase in intracellular Ca^2+^ in N41 cell line. Without compound application, no changes in Ca^2+^ influx could be observed (*n* = 10). Compounds were added to cells at the time points indicated by the arrow and *n* indicates number of the single cells. Experiments were performed in 400 s. The total number of cells was collected in five independent experiments. Values represent mean ± SEM.

To investigate the involvement of TRPs in the 3-T_1_AM stimulatory effects in Ca^2+^ regulation, we monitored time-dependent changes in intracellular free Ca^2+^ levels ([Ca^2+^]_i_) in single-cells. 3-T_1_AM (10 μM) increased the f_340nm_/f_380nm_ ratio from 0.70 ± 0.01 to 0.77 ± 0.05; (*n* = 10) in GT1-7, from 0.70 ± 0.008 to 0.84 ± 0.03 (*n* = 15) in N39 cells and from 0.70 ± 0.009 to 1.88 ± 0.03; (*n* = 15; ^***^p < 0.001) in N41 cells (Figure 5). In untreated controls, this ratio remained constant at 0.70 ± 0.01 in GT1-7 cells (*n* = 10), 0.70 ± 0.009 in N39 cells (*n* = 10) and 0.70 ± 0.01 in N41 cells after the same period (*n* = 15) (Figure 5). It should be noted that the strongest increase of Ca^2+^ concentration was detected in the N41 cells (*p* ≤ 0.0001) which also has the highest expression level of adrenergic receptors and TRPs.

In the next step, we evaluated 3-T_1_AM effects on whole-cell currents of N41 cells to determine if increases in their magnitude underlie rises in plasma membrane Ca^2+^ influx in this cell line. At−60 mV, 10 μM 3-T_1_AM increased inward currents from −14.38 pA/pF to −60.78, which are attributable to Ca^2+^ influx because of the internal Ca^2+^ free solution. At +130 mV, outward rectifying currents strongly increased from 83.71 to 177.38 pA/pF in the presence of 3-T_1_AM.

### 3-T_1_AM mediates rises in Ca^2+^ influx and whole-cell currents through TRPM8 activation

**Figure 6:**
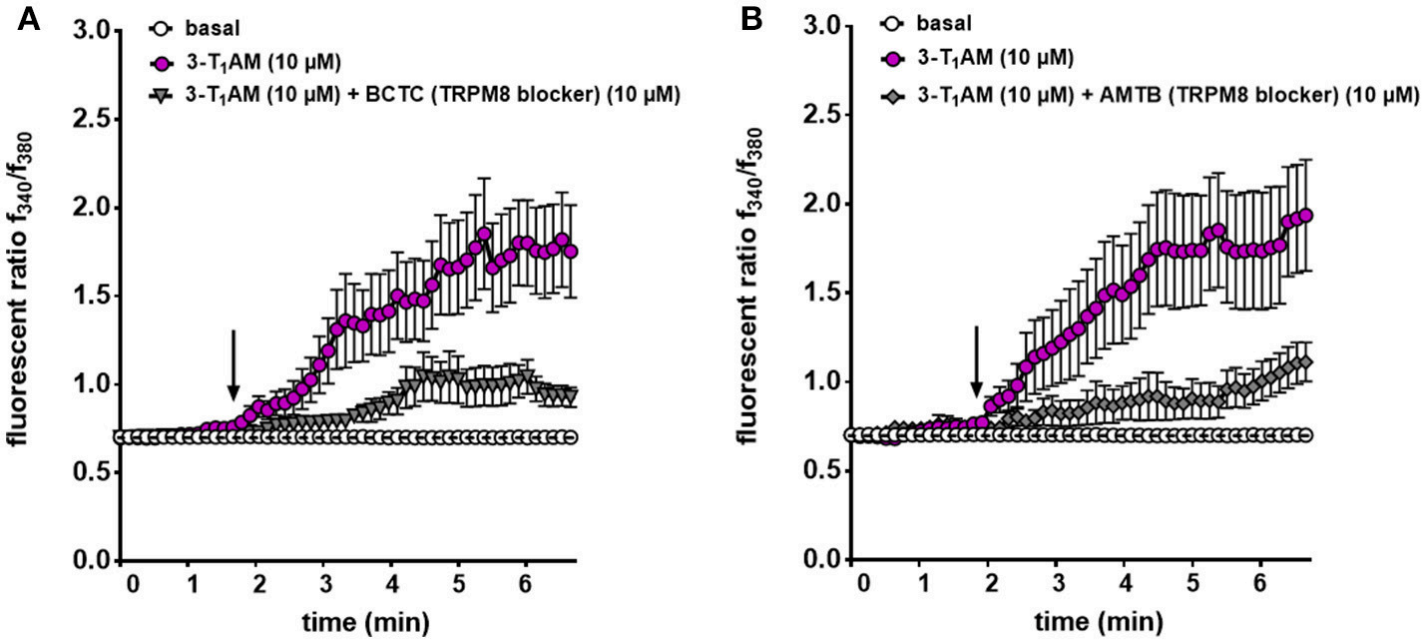
TRPM8 mediates 3-T_1_AM-induced Ca^2+^ response in N41 cell line. Cells were pre-incubated with inhibitors (10 μM AMTB or 10 μM BCTC) 30 min before the measurement. Stimulation was performed with 10 μM 3-T_1_AM and Ca^2+^ influxes were measured (*n* = 15–19) with and without the inhibitors. **(A)** 3-T_1_AM increased Ca^2+^ influx and pre-incubation with BCTC significantly suppressed this effect. **(B)** AMTB showed the similar inhibitory effect on 3-T_1_AM-induced Ca^2+^ influx. Experiments were performed in 400 s. Compounds were added to cells at the time points indicated by the arrow and n indicates number of the single cells. The total number of cells was collected in 5 independent experiments. Values represent mean ± SEM.

Previous studies demonstrated that 3-T_1_AM affects TRPM8 activation at a constant temperature in different cell types (21, 23). To validate that the Ca^2+^ increase stems from an increase in TRPM8 channel activity, N41 cells, which had the maximum response to 3-T_1_AM stimulation, were exposed for 30 min to 10 μM BCTC, followed by bath supplementation with 10 μM 3-T_1_AM. Under these conditions, the TRPM8 channel blocker abolished a 3-T_1_AM-induced Ca^2+^ rise. More specifically, the f_340nm_/f_380nm_ ratio decreased from 1.81 ± 0.04 to 0.93 ± 0.02 in the presence of BCTC (*n* = 15) (*p* ≤ 0.001) (Figure 6A and Supplemental Figure 5).

**Figure 7:**
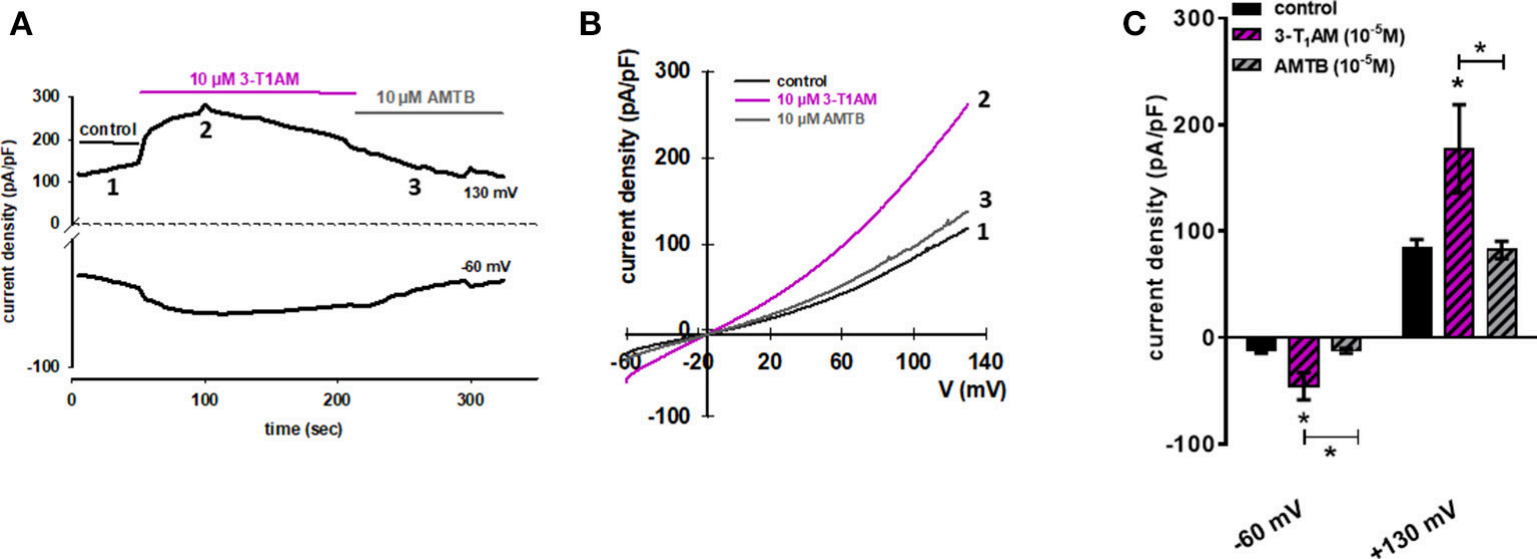
3-T1AM activates whole-cell currents in N41 cell line. **(A)** Time course of whole-cell currents at −60 mV (lower trace) and 130 mV (upper trace) showing the current activation by 10 μM 3-T_1_AM (left). **(B)** Original traces of 3-T_1_AM activated current responses to voltage ramps from −60 mV up to +130 mV (right). Currents are shown before application (labeled as 1) and during application of 1 μM 3-T_1_AM (labeled as 2) and in the presence of 10 μM AMTB (labeled as 3). **(C)** 10 μM 3-T_1_AM increased inward and outward whole-cell channel currents in N41 cells and this effect was strongly suppressed in the presence of 10 μM AMTB. Currents are shown before application (control), during application of 10 μM 3-T_1_AM and in the presence of 10 μM AMTB (*n* = 10). Whole-cell currents were recorded using step and ramp protocols involving voltage steps of 10 mV ranging between−60 to +130 mV for 400 ms. The currents were normalized to capacitance to obtain current density (pA/pF). Statistical significance was determined by one-way ANOVA, comparing the basal current density (pA/pF) against 3-T_1_AM and AMTB. Data are the mean ± SEM of at least 10 independent experiments; *p ≤ 0.05.

Our previous study demonstrated the inverse association between TRPM8 and TRPV1 induced by 3-T_1_AM (21, 22). Different observations showed BCTC acts as a non-specific TRPV1 inhibitor (45, 46). Here, we also demonstrated the high gene expression of TRPV1 in murine hypothalamic cell lines. To rule out the involvement of TRPV1 in the 3-T_1_AM-induced intracellular Ca^2+^ response, we used AMTB as a high selective TRPM8 blocker and capsazepine (CPZ) as a specific TRPV1 blocker. In the presence of 10 μM AMTB, the f_340nm_/f_380nm_ ratio decreased from 1.83 ± 0.08 to 1.12 ± 0.05 (Figure 6B), whereas 10 μM CPZ had no significant inhibitory effect on 3-T_1_AM-induced intracellular Ca^2+^ response (Supplemental Figure 6). As AMTB suppressed a 3-T_1_AM-induced Ca^2+^ increase, we validated this effect by determining if this inhibitor influenced underlying whole-cell currents. In the presence of 10 μM AMTB, inward currents decreased to −14.61 pA/pF and outward currents decreased to 82.40 pA/pF (Figure 7). Considering all these findings, 3-T_1_AM increased intracellular Ca^2+^ concentration and whole-cell currents in mouse hypothalamic cells, thus confirming similar effects in other cell types.

## Discussion

Administration of 3-T_1_AM in mice results in reversible effects such as reduction of body temperature, cardiac output, and the respiratory quotient along with anapyrexia and hyperglycemia (1, 47). There is evidence that 3-T_1_AM accumulates in the hypothalamic nuclei (7, 6). The aim of this study was to explore the underlying mechanism behind the stimulatory effect of this thyroid hormone metabolite in selected hypothalamic regions.

### 3-T_1_AM-induced signalosome activates PVN neurons of C57BL/6 mice

Within the hypothalamus, PVN is one of the most extensively studied nuclei and is playing a pivotal role in the control of fluid homeostasis, lactation, cardiovascular regulation, feeding behavior, nociception and response to stress (11). It has already been shown that the direct injection of 3-T_1_AM into the lateral ventricle of male mice leads to the activation of neurons in the anterior commissural nucleus of hypothalamus (48). Here, we observed that intraperitoneal injection of 3-T_1_AM results in the activation of PVN neurons in C57BL/6 mice (Figure 1). Recently, Gachkar et al. showed that 3-T_1_AM-induced hypothermia is due to vasodilation, which is not directly induced in the veins. In this study they suggested that 3-T_1_AM might induce the tail vasodilation through central action in male mice (14). Here, we investigated the loci in the brain, which are known to be involved in the regulation of the body temperature through non-shivering and shivering thermogenesis (PAG and DMH) and heat dissipation like vasodilation [VTA, (49)]. The result of this study demonstrated no differences in c-FOS activation in these areas (Figure 1B).

Previous studies detected no effect of 3-T_1_AM metabolites such as 3-iodothyroacetic acid and iodine-free thyronamine on cardiac output or thermoregulation (50, 51). Nonetheless, whether 3-T_1_AM directly activates the PVN neurons or causes the activation of neuronal projections from other nuclei is uncertain. Moreover, the exact cellular mechanism initiated by 3-T_1_AM once it reaches in the hypothalamic nuclei is yet to be discovered.

### 3-T_1_AM slightly stimulates Gα_s_ signaling in murine hypothalamic cell lines

Aminergic receptors are largely expressed in the hypothalamus and play a substantial role in various regulatory responses, such as metabolic regulations (52). These receptors are known 3-T_1_AM targets and previous studies assumed that pharmacological effects of this thyroid hormone metabolite are attributable to aminergic receptor signaling (1, 16–19). It has been reported that 3-T_1_AM enhances Gα_s_ signaling in response to ISOP co-stimulation at ADRB2 (17) and activates G_i/o_ signaling combined with NorEpi of the ADRA2A in transfected HEK293 cells (16). Here, we could not detect the activation of G_i/o_ in response to the 3-T_1_AM in murine hypothalamic cell lines (Figure 3B). Nevertheless, N39 and N41 showed an FSK-amplified Gα_s_ signal due to 3-T_1_AM stimulation (Figure 3B). The low GPCR expression rate could be the reason for the lack of strong Gα_s_ in these cells (Figure 2). The absence of G_i/o_ signal through 5-HT1b could be explained by dimerization with TAAR1 (19). It was shown that 3-T_1_AM is capable of inducing a clear G_i/o_ signal in HEK293 cells overexpressing 5-HT1b. However, co-expression of 5-HT1b with TAAR1 abrogated 3-T_1_AM-induced cAMP signaling (19). Since the three hypothalamic cell lines all co-express TAAR1 and 5-HT1b, the G_i/o_ signal of 5-HT1b via 3-T_1_AM stimulation is probably disrupted. The FSK stimulated N39 and N41 cells showed a significant increase in cAMP concentration after 3-T_1_AM challenge (Figure 3B). Besides its own adenylyl cyclase activating property, FSK stimulation additionally favors activation of adenylyl cyclase through Gα_s_ (44, 53, 54). Here, we concluded that 3-T_1_AM induces a weak Gα_s_ signal in N39 and N41 cells.

### 3-T_1_AM activates TRPM8 channel in murine hypothalamic cell lines

Ca^2+^ mobilization in different cell types plays an essential role in the modulation of c-FOS gene expression (55, 56). In neurons, Ca^2+^ influx is a prerequisite for activation of the ERK/MAPK pathway. It is known that c-FOS activation reflects summation or integration of this Ca^2+^ dependent-neuronal activity (57, 58). Moreover, Ca^2+^ acts synergistically with cAMP to activate c-FOS transcription (59). To test whether 3-T_1_AM induces an increase of intracellular Ca^2+^ in hypothalamic cell lines, we evaluated ion channel activation induced by 3-T_1_AM.

It is known that GPCR activation induces an increase of intracellular Ca^2+^ concentration through different pathways (60–63). Recent studies demonstrated the co-expression of GPCRs and TRPs in a variety of cell types (31). Different signaling intermediates such as adaptor proteins, kinases, and lipid metabolites functionally link GPCRs to TRPs (30, 64).

Various TRPs play a pivotal role in the mechanisms that are involved in energy metabolism and temperature adaptation (26, 65, 66). Some recent studies reported that activation of warm-sensitive TRPM2 leads to a similar thermoregulatory response as the one observed in mice after systemic administration of 3-T_1_AM (67). Previously, we showed that 3-T_1_AM acts as a cooling agent to directly affect TRPM8 activation in different cell types (21, 22). In rat thyrocyte, 3-T_1_AM induced Ca^2+^ responses similar to specific TRPM8 agonists such as menthol (68). In ocular cells, 3-T_1_AM evoked Ca^2+^ mobilization and increases in whole-cell currents, a stimulatory effect that could be specifically attenuated in the presence of specific TRPM8 blocker (21, 22). The result of this study showed that 3-T_1_AM induces intracellular Ca^2+^ increase through the TRPM8 channel in murine hypothalamic cell lines. Finding the functional link between 3-T_1_AM and this specific TRP channel in hypothalamic cell lines is relevant since there are indications that the TRPM8 channel regulates energy metabolism in different tissues and plays a crucial role in thermoregulation (69, 70). TRPM8 stimulation, for instance, induces mitochondrial activation and heat production in brown adipocytes. Chronic TRPM8 agonist administration enhances the energy metabolism in brown adipocytes and prevents obesity in mice (69). In skeletal muscles, TRPM8 activation by dietary menthol improves energy metabolism through Ca^2+^-dependent upregulation of the peroxisome proliferator-activated receptor-γ coactivator 1α (PGC1α) which is involved in the mitochondrial function (70). Recently, it has been demonstrated that TRPM8-deficient mice develop late-onset obesity and metabolic dysfunction at moderate cooling, suggesting the importance of TRPM8 in the coupling between thermoregulation and energy homeostasis. Nevertheless, *Trpm8*^−/−^ mice exhibit a remarkable decrease of core body temperature due to increased tail heat loss (71) which is in contrast to the 3-T_1_AM stimulatory effect on TRPM8 found in our *in vitro* electrophysiological observations. Therefore, we investigated the possible effect of 3-T_1_AM on TRPV1 as another known thermo-sensitive TRP channel as it has been shown that there is an interplay between TRPV1 and TRPM8 (72).

Previous studies demonstrated the co-expression of TRPV1 with TRPM8 in different cell types including rat hippocampal neurons, intralobar pulmonary arteries, aorta, neuroendocrine tumor cells, retinoblastoma cells, uveal melanoma cells, and corneal cells (36, 73–76). Here, we showed the co-expression of these two thermo-TRP channels in three different hypothalamic cell lines. It is well established that there is a cross talk between TRPM8 and TRPV1 channels in various tissues. For instance, TRPM8 agonist blocks the mechanical and heat hyperalgesia caused by TRPV1 activation (77, 78). It was also demonstrated that icilin, a specific TRPM8 agonist, attenuates TRPV1-dependent calcitonin gene-related peptide release in the colon and is suggested as a promising therapeutic target for the treatment of colitis (79). The interdependence of the TRPM8 and TRPV1 ion channel function as well as the role of both channels in thermo-regulation have raised the question as to which TRP channel is the main target of 3-T_1_AM in hypothalamic cell lines. The results of this study clearly showed that the effect of 3-T_1_AM were not attributable to TRPV1 since only the specific TRPM8 blocker (AMTB) could strongly inhibit the 3-T_1_AM-induced Ca^2+^ influx and whole-cell current.

Collectively, the results of this study demonstrated the Ca^2+^ signal transduction pathways induced by 3-T_1_AM and provided evidence of TRP channel modulation via this TH derivative in hypothalamic cell lines. Characterization of the intracellular signaling cascade induced by 3-T_1_AM might explain the underlying mechanism behind the profound physiological effects of this metabolite.

## Author contributions

HB, NK, and JB designed the study. JB, SJ, and NK performed the experiments and analyzed the data. SJ, CH, and JM performed the mouse studies. NK, JB, SM, and HB wrote and edited the manuscript. CH, JM, and MR discussed data and edited the manuscript. All authors approved the manuscript.

### Conflict of interest statement

The authors declare that the research was conducted in the absence of any commercial or financial relationships that could be construed as a potential conflict of interest.
