# Supplementary material for: 3-Iodothyronamine Activates a Set of Membrane Proteins in Murine Hypothalamic Cell Lines
Source: Front Endocrinol (Lausanne). 2018 Sep 11;9:523. doi: 10.3389/fendo.2018.00523 (PMC6161562; doi:10.3389/fendo.2018.00523)
Supplement: Supplementary file 1 [file Data_Sheet_1.pdf]

Supplemental material

3-Iodothyronamine activates a set of membrane proteins in murine hypothalamic cells

Julia Bräunig[1], Stefan Mergler[2], Sabine Jyrch[1], Carolin S. Hoefig[3,4], Mark Rosowski[5], Jens Mittag[4,6], Heike Biebermann[1] and Noushafarin Khajavi[1]

[1]Charité – Universitätsmedizin Berlin, corporate member of Freie Universität Berlin, Humboldt-Universität zu Berlin, and Berlin Institute of Health; Berlin, Germany; Institute of Experimental Pediatric Endocrinology, Augustenburger Platz 1, D-13353 Berlin, Germany
[2]Klinik für Augenheilkunde, Charité – Universitätsmedizin Berlin, corporate member of Freie Universität Berlin, Humboldt-Universität zu Berlin, and Berlin Institute of Health, Augustenburger Platz 1, 13353 Berlin, Germany
[3]Institute of Experimental Endocrinology, Charité - Universitätsmedizin Berlin, Corporate Member of Freie Universität Berlin, Humboldt-Universität zu Berlin, and Berlin Institute of Health, Berlin, Germany
[4]Karolinska Instituet, Department of Cell & Molecular Biology, Stockholm, Sweden
[5]Institute of Biotechnology, Department Medical Biotechnology, Technical University of Berlin, Berlin, Germany.
[6]University of Lübeck - Center of Brain Behavior and Metabolism, Lübeck, Germany

Supplemental figure 1

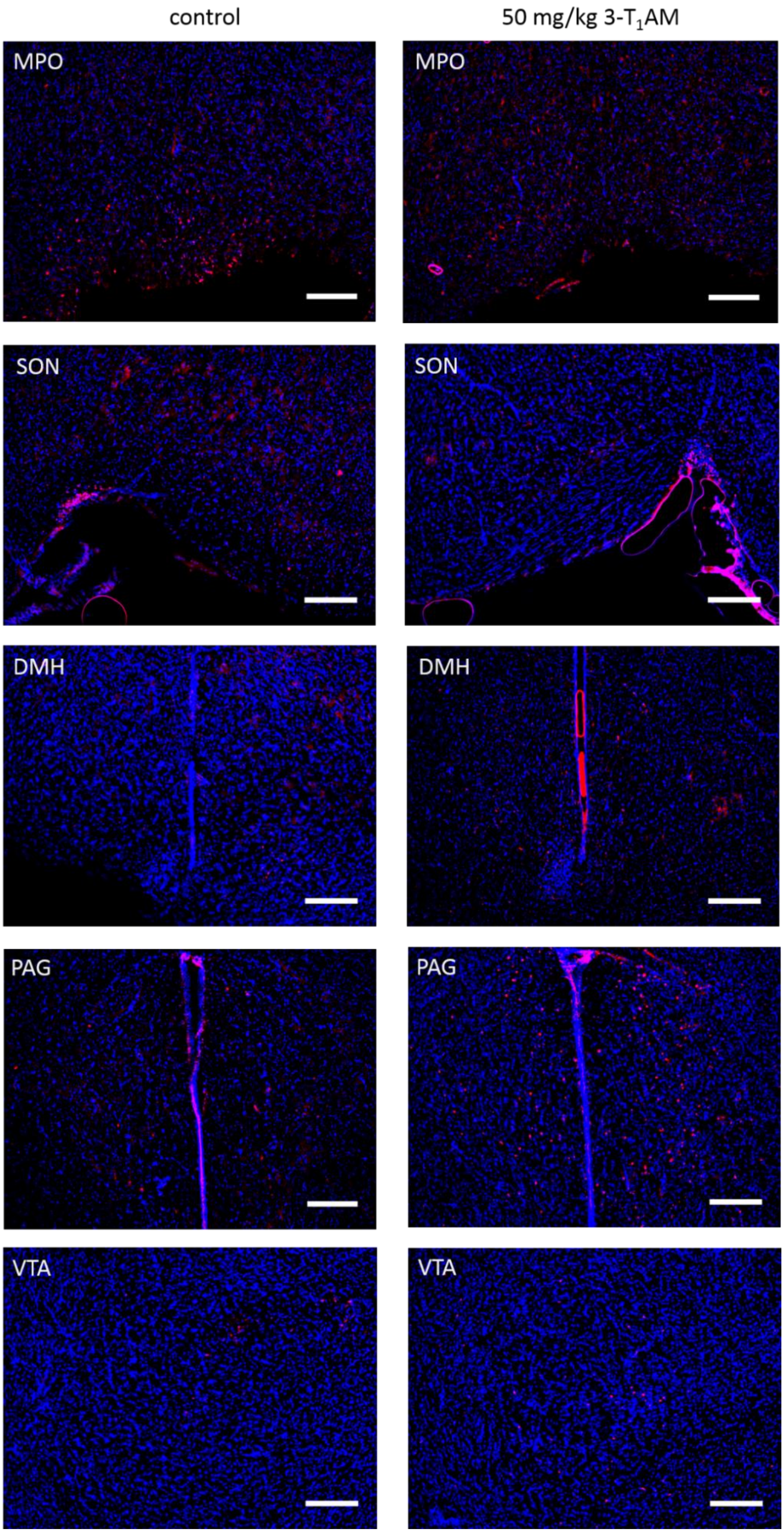

Supplemental figure 1: 3-T_1AM stimulation had no effect on c-FOS staining in the medial preoptic area (MPO), the supraoptic nucleus (SON), the dorsolmedial nucleus of the hypothalamus, the periaqueductal gray (PAG) and the ventral tegemental segment (VTA). After intraperitoneal injection of either 3-T_1AM or solvent (60% DMSO/ 40% PBS), brains of the C57BL/6J mice were frozen, cryosectioned and stained against c-FOS (pink) and DAPI (blue) (n=3). All pictures were taken with a 20× objective. The scale bar indicates 200 µm.

Supplemental figure 2

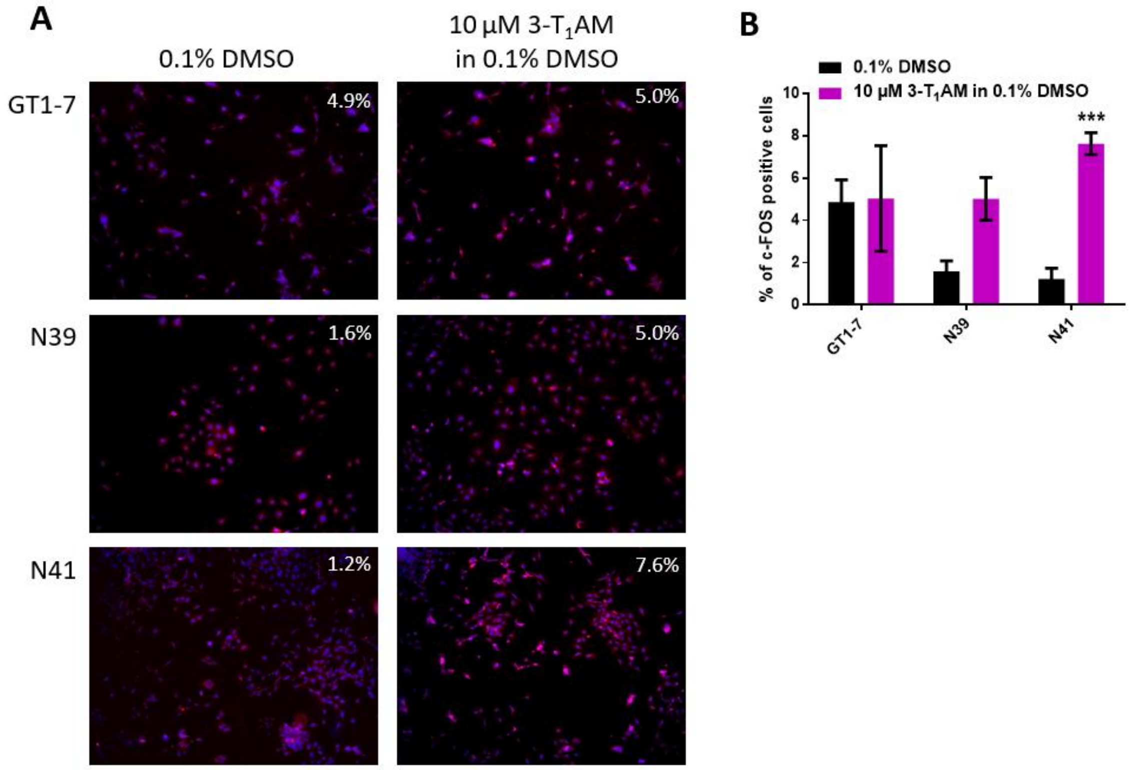

Supplemental figure 2: 3-T$_1$AM induces c-FOS activation in N41 cells.
(**A**) Cells were seeded on poly-L-lysine coeated glas slides, after 48 h of standard cultivation, cells were incubated with AdvancedMEM and 0.1% DMSO or 10 µM 3-T$_1$AM for 1h. After fixation cells were stained with c-FOS (pink) and DAPI (blue) (**B**) Percentages of c-FOS positive cells in the hypothalamic cell lines GT1-7, N39 and N41. For statistics, unpaired t test with Welch's correction was performed. Data are the mean ± SEM of 3 independent experiments; ***$p \leq 0.001$.

Supplemental figure 3

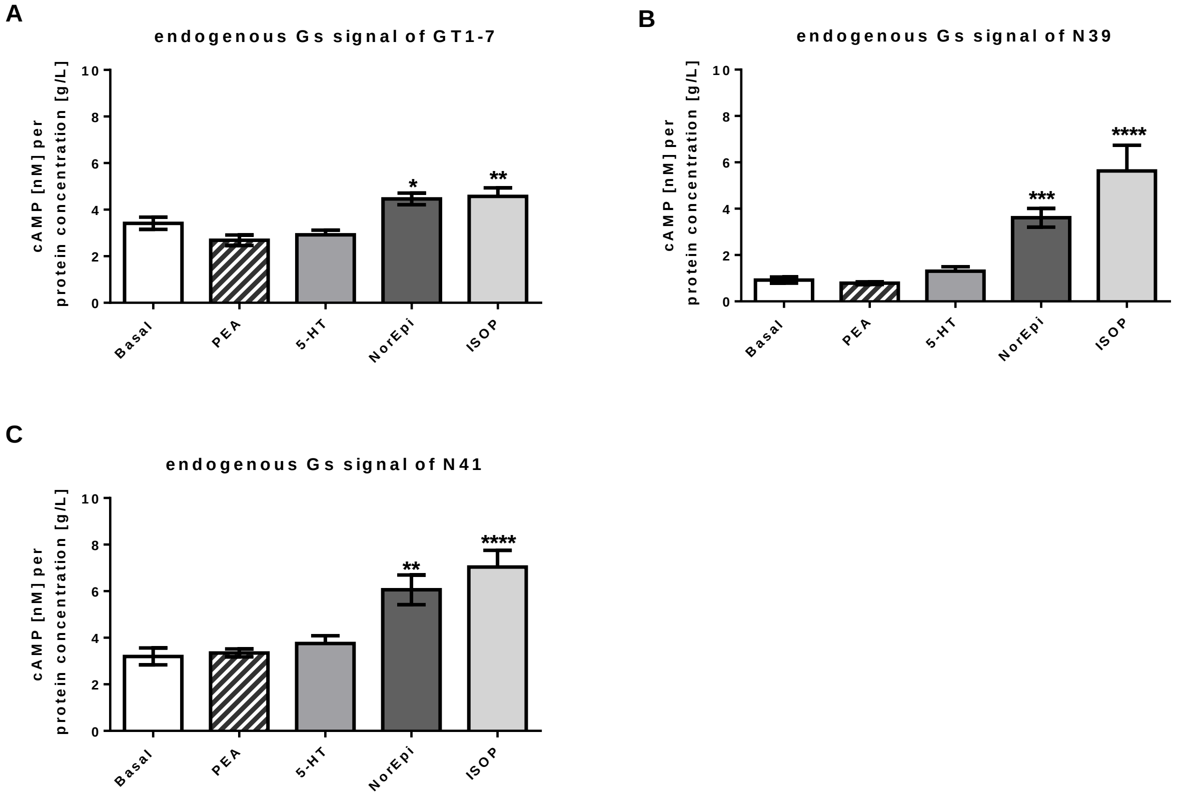

Supplemental figure 3: NorEpi and ISOP induce cAMP accumulation in GT1-7, N39 and N41 cells. For $G\alpha_s$, the cAMP content was measured via AlphaScreen technology. **(A)** GT1-7, **(B)** N39 and **(C)** N41 cells were stimulated with stimulation buffer, PEA, 5-HT, NorEpi or ISOP in a concentration of 10^{-5} M for 45 min. For statistics, a two-way ANOVA was performed, followed by a Sidak correction. Data are the mean ± SEM of 3-4 independent experiments measured in triplicates; *$p \leq 0.05$, **$p \leq 0.01$, ***$p \leq 0.001$, ****$p \leq 0.0001$.

Supplemental figure 4

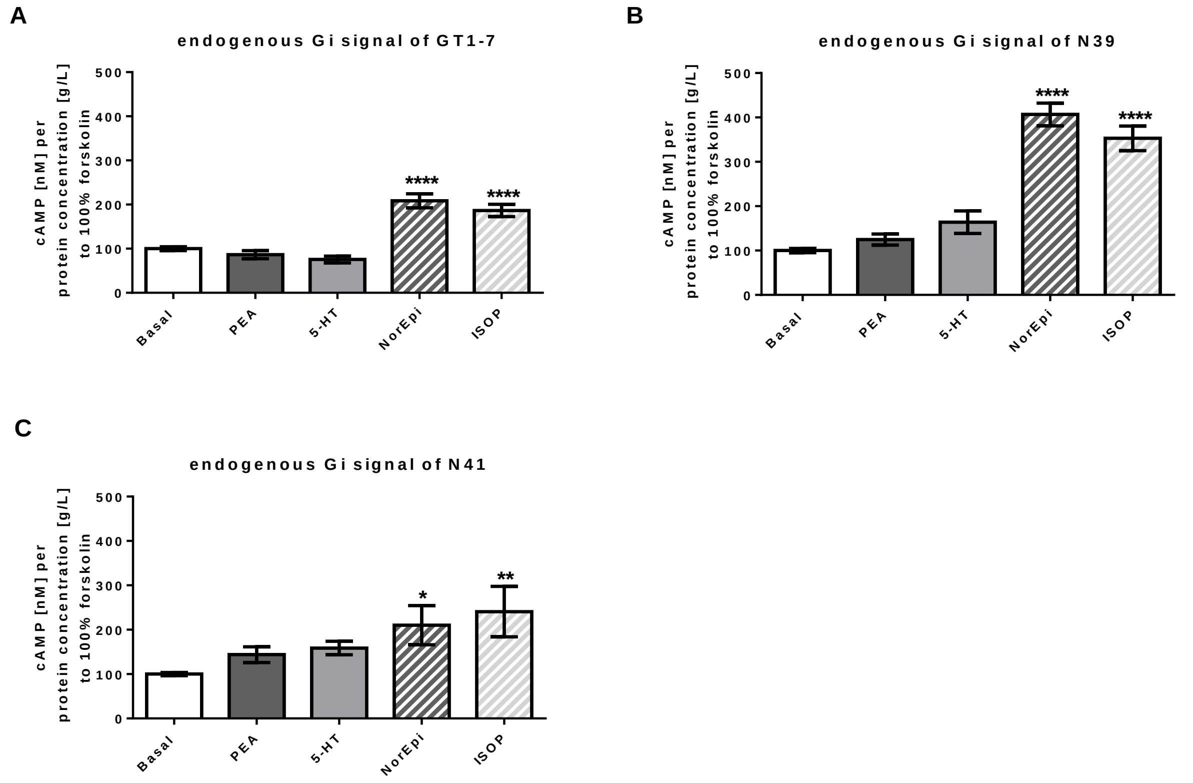

Supplemental figure 4: Stimulation with aminergic ligands had no effect on $G_{i/o}$ signaling. For $G_{i/o}$, the cAMP content was measured via an AlphaScreen Kit. **(A)** GT1-7, **(B)** N39 and **(C)** N41 cells were co-stimulated with forskolin and either stimulation buffer, PEA, 5-HT, NorEpi or ISOP in a concentration of 10^{-5} M for 45 min. For statistics, a two-way ANOVA was performed, followed by a Sidak correction. Data are the mean ± SEM of 3-4 independent experiments measured in triplicates; *$p \leq 0.05$, **$p \leq 0.01$, ***$p \leq 0.001$, ****$p \leq 0.0001$.

Supplemental figure 5

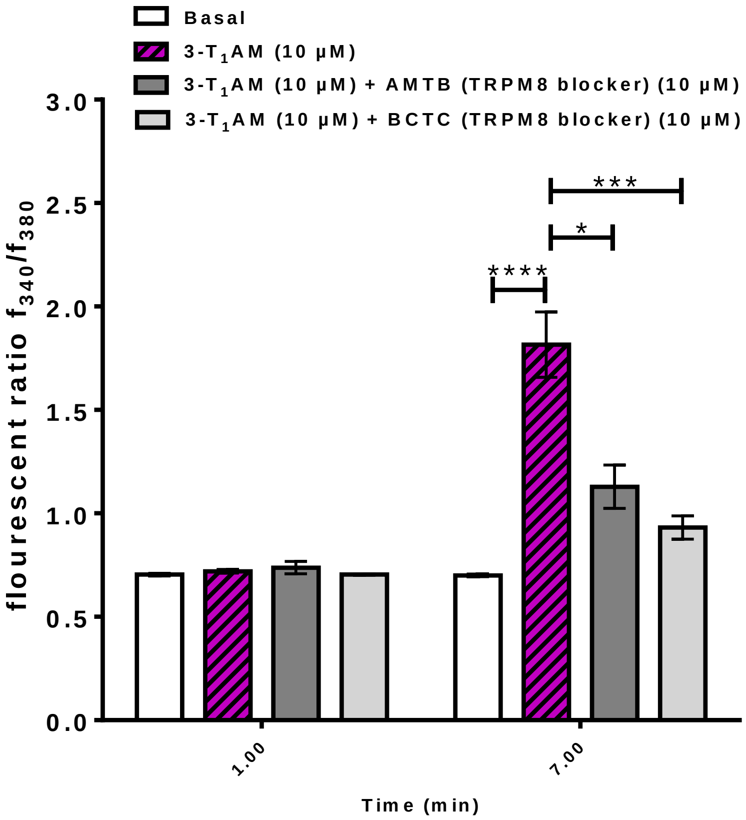

Supplemental figure 5: TRPM8 is strongly involved in 3-T_1AM-induced Ca^{2+} influx. Summary of the experiments with 3-T_1AM and different blockers. 10 µM 3-T_1AM significantly increased f_{340nm}/f_{380nm} ratio compare to the control sample. Stimulatory effect of 3-T_1AM on Ca^{2+} influx was significantly blunted in the presence of 10 µM BCTC or 10 µM AMTB. Statistical significance was determined by an unpaired t test with Welch's correction, comparing f_{340nm}/f_{380nm} ratio between 1 min and 7 min of measurement with and without agonists. Data are the mean ± SEM of 5 independent experiments. Asterisks (*) indicate differences of f_{340nm}/f_{380nm} ratio between different time points; * $p \leq 0.05$, ** $p \leq 0.01$, *** $p \leq 0.001$, **** $p \leq 0.0001$.

Supplemental figure 6

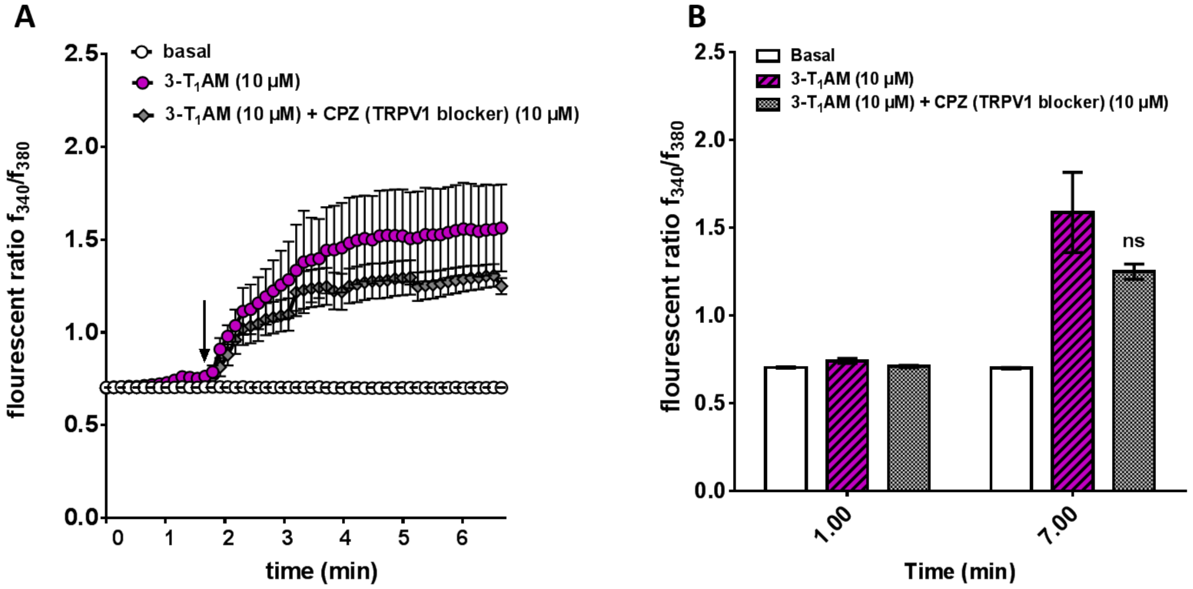

Supplemental figure 6: Capsazepine (CPZ) has no inhibitory effect on 3-T$_1$AM-induced Ca^{2+} influx. The specific inhibitor of TRPV1, CPZ was used to rule out the involvement of TRPV1 in 3-T$_1$AM-induced Ca^{2+} influx. **(A)** 10 µM 3-T$_1$AM significantly increased f_{340nm}/f_{380nm} ratio, while pre-incubation with 10 µM CPZ had no inhibitory effect in this response *(n = 16)*. **(B)** Summary of the experiments with 3-T$_1$AM and blocker. Statistical significance was determined by an unpaired t test with Welch's correction, comparing f_{340nm}/f_{380nm} ratio between 1 min and 7 min of measurement with and without agonists. Data are the mean ± SEM of 3 independent experiments. Asterisks (*) indicate differences of f340nm/f380nm ratio between different time points; * $p \leq 0.05$, ** $p \leq 0.01$, *** $p \leq 0.001$, **** $p \leq 0.0001$.

Supplemental table 1

	**forward**	**reverse**	**efficiency**
Pgk1	5′-TCGTGATGAGGGTGGACTTC	5′-CCAGGTGGCTCATAAGGACA	*1.70*
Taar1	5′-AATGATGTCCGTGCTTCCCT	5′-ATGACCAGACACCCCAGAAG	*1.50*
5-Ht1b	5′-GTGAACACCGACCACATCCT	5′-GGAGTCGGTTATCAGCTGGG	*1.85*
Adra2a	5′-CACGCTCGTCATCCCTTTCT	5′-ACTCGATGGCCTGTGTGATG	*1.66*
Adrb1	5′-ACGCTCACCAACCTCTTCAT	5′-GCAATGACACACAGGGTCTC	*1.44*
Adrb2	5′-AGAGCCTGCTGACCAAGAAT	5′-CACGATGGAAGAGGCAATGG	*1.66*
Trpm1	5′-GTGAGCACTGGTGTCGTCA	5′-CTCAGAGGGTTGGACATGGT	*1.95*
Trpm2	5′-CTTGGACCCGGAGAAGAACTG	5′-TCGGGAATCCATGAGCTAAGG	*1.80*
Trpm3	5′-GAACTCCAGCCCAAACTCAAG	5′-GGGGCGATACCTATGGTACATAT	*1.95*
Trpm4	5′-AGCACAGCAACTTTCTCCGG	5′-CACCGACACCACCAAGTTTG	*1.40*
Trpm5	5′-ACATCCACCAAGATCCGTGT	5′-TCCCTGAATGTTGCCCTCAT	*1.39*
Trpm6	5′-GACCGTCAAGAACAAGGAGC	5′-CGTAGAATCCCTCCATCCTCC	*1.97*
Trpm7	5′-GAGTTCCTGTGGTGGCTTTG	5′-CACAACAACTGGAACTGGGG	*1.45*
Trpm8	5′-GAGCAAGACAAGGACAACTGG	5′-GTCCTTATGAGAGCCGTGAAC	*1.95*
Trpv1	5′-CTGAAGTGCATGAGGAAGGC	5′-AGTTCACCTCATCCACCCTG	*1.93*

Supplemental table 1: Primer pairs for reference gene, GPCRs and TRP channels and their determined amplification efficiency
